# Addressing Challenges in Endometriosis Pain Communication Between Patients and Doctors: The Role of Language

**DOI:** 10.3389/fgwh.2021.764693

**Published:** 2021-11-15

**Authors:** Stella Bullo, Annalise Weckesser

**Affiliations:** ^1^Department of Languages, Information and Communications, Faculty of Arts and Humanities, Manchester Metropolitan University, Manchester, United Kingdom; ^2^Centre for Social Care and Health Related Research, Faculty of Health, Education and Life Sciences, Birmingham City University, Birmingham, United Kingdom

**Keywords:** pain, communication, language, metaphor, endometriosis

## Abstract

**Introduction:** In the context of the complex medical, social, and economic factors that contribute to endometriosis diagnosis delay and its consequent impact on quality of life, this report focuses on patient-practitioner pain communication and examines the role of language in doctor-patient communication. Our study explored what patients and doctors consider challenging and effective in endometriosis pain communication. It further examined what commonly used metaphors by patients could be suggestive, or not, of endometriosis to doctors.

**Method:** A United Kingdom-based qualitative (open-ended question) survey with women with endometriosis (n131) and semi-structured telephone interviews with general practitioners (GPs) (n11). Survey and interview data were analyzed thematically.

**Results:** Both women and GPs reported the Numeric Rating Scale (NRS) to be insufficient as a standalone tool for communicating endometriosis related pain. Both also found descriptions of the quality, location, and impact on daily life of pain to more effective means of communicating pain symptoms. When presented with common metaphorical expressions surveyed women used to describe their pain, not all GPs recognized such metaphors as indicative of possible endometriosis. Further, some GPs reported some of the expressions to be indicative of other pathologies.

**Conclusion:** Findings reveal the importance of language in pain communication and the need for additional tools to help women and doctors find the most effective way to communicate the experience and elicit appropriate investigative care. They also show the need for further investigation into how metaphor can be effectively used to improve patient-practitioner communication of endometriosis related pain.

## Introduction

The impact of endometriosis on quality of life has been widely reported (1), concluding that it is multidimensional, complex, and pervasive causing women to feel a sense of powerless and lack of control (2, 3). Such disempowering effect have been attributed to pain normalization and dismissal, which are considered a potential cause of diagnosis delay (4). Additionally, perceived deficiencies in communication to effectively describe pain symptoms in early consultations have been seen to play a role in delaying diagnosis (5) with consequent impact on the physical, mental, and social well-being of women with the condition (6).

The difficulties posed for managing possible endometriosis in primary care have been recently recorded in qualitative research (7). Amongst the challenges for endometriosis diagnosis identified, studies have highlighted the complexity of endometriosis pain types, namely cyclical, functional, and chronic (8) with nociceptive and neuropathic pain characteristics (9). Further to this, the perception of endometriosis pain may also be influenced by other factors such as physical stress, hormonal cycles, and pain-coping strategies (10). Indeed, research suggests the psychological distress caused by endometriosis can heighten the sensitivity to pain (11). Therefore, pain assessment tools should consider not only severity, normally measured through the Numeric Rating Scale (NRS), but also qualitative aspects (12) as a potential way to understand its underlying mechanisms and its interference with physical and emotional aspects of quality of life. Pain assessment tools considering such aspects have indeed been devised by endometriosis specialists (8), but these are mostly used post diagnosis in order to inform treatment. Thus, pre-diagnosis pain complaints are not always systematically assessed through multifactor tools in early consultations.

The communication challenges posed by the lack of visibility, physical manifestations, and experiential understanding of invisible pain, as is the case with endometriosis, leads patients to rely on metaphorical language (13, 14). Metaphor in language refers to the conceptualization of an abstract concept, or domain, in terms of another, usually more a concrete, experiential, and embodied domain, resting on mappings and entailments connecting both (15). However, the nuances of such conceptual frameworks and their manifestation in language use, along with the assumptions brought to the clinical communication by both patients and doctors may pose a challenge for successful communication to take place. A mismatch in assumptions through the use of unconventional language or descriptors that fall outside the expected frames of reference can indeed result in miscommunication. For women living with endometriosis, this can potentially lead to misdiagnosis, pain dismissal, or normalization (5), and subsequent negative impact on quality of life.

Studies of metaphorical language describing chronic pain, including endometriosis, are abundant [e.g., (5, 16)]. However, they mostly focus on patients' metaphorical descriptions of chronic pain, but do not address doctors' perspectives on what such use of metaphors suggests for diagnosis or referrals. Thus, in this study, we aimed to examine what both patients and doctors consider effective and challenging in endometriosis pain communication. Additionally, we explored what common metaphors used by patients can be suggestive, or not, of endometriosis for doctors.

## Methods

A qualitative design, using an open-ended question survey for women with endometriosis and semi-structured telephone interviews with doctors providing care to those with suspected and diagnosed endometriosis, was used.

The Microsoft Forms, internet-based survey, was distributed *via* social media using the Twitter account @EndoLanguage, tagging United Kingdom (UK) based endometriosis support organizations. The main criteria for self-selecting participants were to have a diagnosis of endometriosis, be based in the UK and have a proficient level of English to answer open-ended questions. Consent was obtained before proceeding to the survey page. The survey aimed to identify deficiencies in the communication of endometriosis pain during early consultations. This study reports on responses to the following two open questions:

1. What difficulties did you encounter when communicating pain in early consultations before your diagnosis of endometriosis?
2. Describe your pain; how does your pain feel?

Interviews were conducted with doctors who responded to a call for participants distributed *via* the same social media site and through endometriosis networks using snowballing sampling. A total of 18 UK-based private practice and National Health Service (NHS) GPs (n11) and specialist gynecologists (n7) were interviewed. In this paper, we report only on interviews with GPs in order to identify communication challenges during consultations prior to referral for further investigative care. Table 1 summarizes GPs demographic information.

**Table 1:** GPs demographic information.

**Profession**	**Sex**	**Years practicing**	Special Interest Area(s)^*^
GP1	Female	2.5	National NHS Policy
GP2	Female	20	Gynecology
GP3	Female	13	Occupational Health (private)
GP4	Female	14	Women's Health
GP5	Female	14	Gynecology, Contraception, Sexual Health, Epilepsy, and Gastroenterology
GP6	Female	14	Womens' Health and Sexual Health
GP7	Female	4.5	None
GP8	Female	0.5	None
GP9	Female	3	None
GP10	Female	5.5	Palliative Care and Dermatology
GP11	Female	16	Women's Health and Contraception

* *All but one GP work in public practice for the NHS*.

Interviews were semi-structured and lasted 20–30 min. Consent was obtained prior to the interviews and data have been anonymized as required by ethical protocols. This study reports on responses to the following questions regarding pain communication:

1. How do you assess suspected endometriosis pain?
2. Do you use the 1–10 scale to assess severity of pain? Why or why not?
3. How do patients talk about pain?
4. Have you heard the following expressions or similar ones by patients describing pain?
  a. “as if somebody is repeatedly stabbing me with a large knife”
  b. “as if I've got a balloon inside me pushing on everything”
  c. “like knitting needles being pushed through your abdomen”
  d. “like having a hot poker planted inside your stomach”

Survey data was downloaded in an excel spreadsheet. A total of 131 responses were eligible for analysis. The mean time for diagnosis was 9.3 years. GP audio recorded interviews were transcribed verbatim. Both data sets were imported to NVivo11 for analysis where themes and subthemes were identified and verified by the research team [a linguist (Author 1) and medical anthropologist (Author 2)]. The analysis was conducted in line with Clark's (17) protocol for qualitative research outlining Relevance, Appropriateness, Transparency, and Soundness. Tables 2, 3 summarize the main themes emerging in both datasets.

**Table 2:** Patient views of pain communication themes and sub-themes.

**Main themes**	**Sub-themes**
Pain communication difficulties	Numeric scales restrictive and subjective
	Lack of qualitative tools for pain description
	Lack of enquiry of how pain impacts day-to-day life
	Lack of elicitation of symptoms by doctors
Perceived lack of listening	Perceived lack of active listening
	Lack of empathy
	Dismissal and normalization
Pain described using metaphorical language	Pain as physical damage *via* violent acts or physical actions
	Pain as property of elements

**Table 3:** GP views of pain communication themes and sub-themes.

**Main themes**	**Sub-themes**
Multifactor indicators	NRS markers helpful in combination with other tools
	Impact on life
	Location
	Frequency and duration
	Qualitative descriptors
Individual/contextual factors	Subjective/variable perceptions of severity
	Language barriers
	Taboos in disclosure of certain symptoms
Descriptors of pain	“achy,” “crampy,” “labor-like,” “something you'd put a hot bottle on,” “sharp,” “dull,” “dragging”
	Pain as physical damage metaphors: 5 out of 11 doctors
	Pain as properties of elements: 6 out of 11 doctors

The four metaphorical expressions presented to doctors in interviews (Questions 4a-b above) were a representative selection of those most recurrent in patient survey data. The metaphors were identified by reference to the Pragglejaz Group (18) which works by establishing a contrast between the contextual meaning of an expression with the basic dictionary meaning.

## Results

### Patient Views of Pain Communication

The table below summarizes themes identified in patient survey data.

#### Pain Communication Difficulties

Women reported experiencing difficulties describing pain during pre-diagnosis medical interactions. Such difficulties in pain communication were attributed to various aspects. Firstly, numeric scales offered were seen as too limited to accurately capture the severity and the impact of pain on quality of life (a). Furthermore, patients advocated the facilitation of a descriptive methodology and other tools to describe pain qualitatively (b) and claimed that visual tools would also be helpful in allowing to point to where pain is felt and understand its mechanisms (c). Lastly, women reported wanting doctors to ask specific questions about pain type (and other associated symptoms) (d), as well as to discuss the impact on their day-to-day lives (e), to better elicit and understand pain experiences.

*(a) [Do] not rely on the 1–10 pain scale. My 7 could be someone else's 10. It's not accurate. Listen to us describe how our pain keeps us from doing everyday tasks*
*(b) I did not associate what I was feeling as “pain.” I needed to see a list of feelings in order to do this, a list with words like burning, dragging, squeezing, etc*.
*(c) Maybe have a pic of female anatomy to help show where the organs are in the body so you can point and show where the pain is or feels like it is*
*(d) [A]sk more specific questions about the type of pain and ask about other symptoms*
*(e) [L]et patients talk about how the pain keeps them from living their everyday lives, such as it hurts when I have sex, exercise, go up stairs, stand for too long, etc. Also, give key words and/or examples to describe the pain. “It feels like someone is stabbing me” or “It feels like I'm having contractions before giving birth”*

#### Perceived Lack of Listening

Women reported feeling that doctors do not/or have not actively listened to them when describing symptoms. Many reported feeling rushed, discouraged from talking (f) or even intimidated (g), leading them to perceive that their pain and symptoms are being dismissed or normalized (h, i).

*(f) [A]llow [patients]to talk about it more in depth and in detail. Believe everything being said, don't rush the appointment … Do not discourage the patient from expressing her pain. Give her the freedom to speak freely about what she is going through*
*(g) I find docs intimidating and sometimes lose my words because they are very abrasive and harsh regarding pain*
*(h) Listen more and not easily dismiss me when I tried to explain the pain in detail*
*(i) Stop acting like the pain I'm in is normal. It's not normal to be stuck in bed for 3 days*

#### Pain Described Using Metaphors

Many women used similes, metaphors comparing an abstract concept to a concrete one but the conceptual connection is purposefully marked (e.g., “like,” “as if”) for rhetorical effect (19), to describe their pain. In using such metaphorical language, mostly including violence [e.g., (10)], women attempt to indicate not only quality and severity, but also the physical and psychological impact of their pain (20).

The main sub-theme of metaphorical scenarios women used to describe endometriosis pain make it akin to physical damage that would result from a violent act (j, k). Usually they referred to the action (e.g., stabbing) or the objects that would cause such damage (e.g., knife). The latter is known as metonymy, where the object stands for the action (e.g., “it's a knifey pain”). Other metaphors in this sub-theme also compared pain to a physical action that could also lead to physical damage (e.g., pulling, squeezing) (l).

*(j) as if somebody is repeatedly stabbing me with a large knife*
*(k) like knitting needles being pushed through your abdomen*
*(l) like constant pulling in my abdomen*

The above quotes (j, k) compare pain to actions that would cause physical damage and nociceptive pain (stabbing; piercing). The descriptions are enhanced by metonymical relations (knife and knitting needles) where the objects seen to cause the damage are also qualified in terms of size (large knife) and type (knitting needle). In (j), the conceptual association that such metaphorical and metonymic connections have with endometriosis pain indicate the conceptualization of pain and its mechanisms as sharp, sudden, intermittent and aggressive. The adverb “repeatedly” adds further information about the duration and recurrence of pain. In (k), the conceptual association indicates that pain is less sharp and aggressive, perhaps compromising a smaller area, but equally acute, bothersome and damaging (i.e., intensity) and longer lasting. In both cases, pain is compared to torturing actions inflicted by a violent external entity.

The second main sub-theme of metaphorical descriptions of pain used by women compared it to properties of elements. That is, pain is related mainly to heat (m) or pressure (n).

*(m) like having a hot poker planted inside your stomach*
*(n) as if I've got a balloon inside me pushing on everything*

Metaphorical expressions indicating heat and pressure either in the form of similes or qualifying pain (e.g., burning pain) were very common. The “hot poker” metaphor was highly recurrent in the survey data. This metaphor blends both sub-themes (i.e., pain as physical damage *via* a violent act and as an elemental property) as it compares pain to heat but also to an external pointed (hot) object causing damage. Pressure, both in metaphorical expressions (e.g., “a balloon,” “a volcano about to erupt”) and literally “a lot of pressure,” was also highly recurrent.

### GPs Views of Pain Communication

The table below summarizes themes identified in GP interview data.

#### Multifactor Indicators of Pain Suggesting Endometriosis

Most GPs reported to use the NRS to assess possible endometriosis related pain, but stated that the scale works better when combined with further queries relating to pain and its impact on daily life (o), location (p), and frequency and duration (q). In addition to the NRS, some also reported seeking out qualitative descriptors of pain (r).

*(o) [S]ometimes I get people to scale it for me from 0 to 10. I think then really more describing the impact it has on their life, so they're in bed with it or they miss school because of it or they miss work because of it and it knocks them out for 2 days, over 2 or 3 days every month. It's normally that kind of conversation*.
*(p) [T]he typical one would be lower abdominal pain, so location first of all*.
*(q) I don't know that there's one, sort of, set pattern of pain that would particularly make you think, oh this is endometriosis, other than if it starts initially with a cyclical pattern and then, sort of, you see a progression over months and years of increasing pain*.
*(r) Yes [use NRS], and I try to get them to talk about it in qualitative terms as well. So I will always ask people whether it's a cramping type pain, an aching type pain, a stabbing type pain…but I think it can be quite difficult because some people will be describing pain that isn't cyclical and then that makes it harder*.

#### Individual and Contextual Factors Affecting Pain Communication

Despite using the NRS, many GPs acknowledged the subjectivity and individual variability of the scale (s, t). GPs also reported other aspects that may impact effective communication of pain and symptoms disclosure, such as language barriers when seeing patients who are not fluent English speakers, or perceived taboos around certain topics, including painful intercourse (u) and painful bowel movements (v).

*(s) I don't find it always that helpful to be honest, using a scale, because it's subjective. So what's an eight to me might be a five to someone who's a bit braver*.
*(t) Severity of pain and patients' perception of pain, sort of (depends on) how their minds associate pain with problems, it's hugely variable in the population. So, I don't think severity alone is the key to the diagnosis*.
*(u) Pain on deep intercourse, deep penetration (…) a lot of people don't openly, talk about that. It's only if you specifically ask that people will tell you that*.
*(v) [Discussing pain during bowel movement] sometimes people might think, “Oh it's not ladylike.” They just don't really openly (talk about that)*.

#### Descriptions of Pain

GPs reported that more qualitative descriptors of pain they hear from patients with suspected endometriosis were terms such as: *achy, crampy, labor-like, “something you'd put a hot bottle on,” sharp, dull, persistent, incapacitating*, and *dragging*.

In response to interviewers' questions about metaphors for endometriosis pain (Question 4a-b) used most prominently by women in survey data, some GPs (n5) stated that they recognized the expressions for pain as physical damage (j, k) and would suspect endometriosis. Similarly, some GPs (n6) recognized the metaphors for heat and pressure (m, n). One GP stated some patients use paralanguage, such as pointing at the area or making squeezing or pulling gestures, which they found helpful (w). This is consistent with the metaphors for physical damage caused by physical actions (l) discussed above.

*(w) [P]eople will make squeezing motions with their hands, or point, often I think probably subconsciously to the area of their body that is affected by the pain they're trying to describe verbally. Those descriptions, they're helpful probably in trying to exclude things*.

Some GPs (n3) indicated that such metaphors would make them suspect non-endometriosis pathologies and would possibly investigate other alternative conditions first:

*(x) [I]f someone was describing something as, I don't know, as very intermittent, very short, burning, stabbing pain, that to me is much more a description of a neuropathic pain than a gut or pelvic organ pathology*.
*(y) I think I would associate those words more with neuropathic type pain, so nerve pain rather than organ pain. I would be thinking more of crampy type pain for example, as endometriosis pain. So, that's very interesting*.
*(z) [W]hen they describe it like this, I'm pretty sure the first thing would be to investigate infection with the urine samples and swabs*.

## Discussion

In patient-practitioner communication of endometriosis related pain, both women and GPs find the NRS to be an ineffective (standalone) tool due to the subjectivity of pain and the inability of such scales to capture qualitative aspects of pain. While many GPs use the tool, they do so in conjunction with additional queries (i.e., on pain duration and frequency, location, descriptors, and impact on daily life) to better understand and assess whether such symptoms are indicative of possible endometriosis. Women want GPs to ask such investigative questions about the nature of their pain experiences (i.e., on pain type, location, descriptors, and impact on daily life). Many, however, feel their pain is normalized and/or dismissed by practitioners who did not adequately listen and seek to understand their symptoms.

Women draw on metaphorical language to better capture and articulate their endometriosis related pain. GPs, however, do not always recognize the metaphors women commonly use as indicative of the condition. In some cases, such metaphors were seen by GPs to be more likely caused by other (often neuropathic) conditions.

A limitation of this study is the relatively small number of GPs (n11) who took part. However, given the well-established challenge of recruiting GPs to qualitative research due to their professional time constraints (21), insights gained from the views of such a group are valuable given their paucity. To our knowledge, this is the first UK-based qualitative study to focus on both patients' and doctors' views on endometriosis pain communication, in particular. The mixed disciplinary background of the research team (a linguist and a medical anthropologist) is also a strength of the study.

Both women and GPs view understanding the impact on the quality of (daily) life as key to effectively communicating endometriosis pain, indicating the need for pre-diagnostic screening that incorporates quality of life considerations as well as psychological impact (22). To date, the only endometriosis-specific quality of life tool, the Endometriosis Health Profile-30, is a post-diagnosis instrument (23). Further research on effective pre-diagnosis patient-practitioner communication of pain indicative of possible endometriosis, which incorporates queries around pain location, quality, timing and duration are also needed. Effective communication of pain is collaborative work between patients and doctors. The use of metaphors can improve practitioner-patient communication [e.g., in relation to other serious health conditions, see (24)] and help patients interpret, accept and adapt to pain as a coping mechanism (20). Therefore, further investigation is needed into the effective use of metaphor to enhance patient-practitioner communication of endometriosis related pain in general practice care as well as into improving GPs' understandings of common metaphors employed by women living with the condition.

## Data Availability Statement

The original contributions presented in the study are included in the article/supplementary materials, further inquiries can be directed to the corresponding author.

## Ethics Statement

The studies involving human participants were reviewed and approved by Birmingham City University Manchester and Manchester Metropolitan University. The patients/participants provided their written informed consent to participate in this study.

## Author Contributions

SB and AW contributed to conception, design of the study, collected database 2, and data analysis. SB collected database 1, data analysis, and wrote abstract. All authors contributed to manuscript.

## Funding

This study was supported by Birmingham City University's Faculty of Health, Education and Life Sciences-Pilot Funding.

## Conflict of Interest

The authors declare that the research was conducted in the absence of any commercial or financial relationships that could be construed as a potential conflict of interest.

## Publisher's Note

All claims expressed in this article are solely those of the authors and do not necessarily represent those of their affiliated organizations, or those of the publisher, the editors and the reviewers. Any product that may be evaluated in this article, or claim that may be made by its manufacturer, is not guaranteed or endorsed by the publisher.
